# Unveiling the hidden risks: albumin-corrected anion gap as a superior marker for cardiovascular mortality in type 2 diabetes: insights from a nationally prospective cohort study

**DOI:** 10.3389/fendo.2024.1461047

**Published:** 2024-11-07

**Authors:** Mingsi Wang, Shu Yang, Jingwen Deng, Dehai Wu, Changzhi He, Guanghua Li, Ying Dong, Yongxiang Zhang, Yilan Li

**Affiliations:** ^1^ The Second Affiliated Hospital of Harbin Medical University, Harbin, China; ^2^ College of Health Management of Harbin Medical University, Harbin, Heilongjiang, China; ^3^ Heilongjiang University of Chinese Medicine, Harbin, China; ^4^ Key Laboratory of Myocardial Ischemia, Ministry of Education, Harbin Medical University, Harbin, China; ^5^ Graduate School, Harbin Medical University, Harbin, Heilongjiang, China

**Keywords:** albumin-corrected serum anion gap, NHANES, diabetes mellitus, cardiovascular disease, mortality

## Abstract

**Aims:**

Hypoalbuminemia can lead to underestimations of the true anion gap levels. There are few data on albumin-corrected serum anion gap (ACAG) status and mortality in the diabetes. The study aimed to examine the association between ACAG and all-cause, cardiovascular, and cancer mortality in type 2 diabetes (T2D) patients.

**Methods:**

Herein, 8,161 diabetic adults were included in the National Health and Nutrition Examination Survey (NHANES) 1999-2018. National Mortality Index (NDI) data were used for determining mortality outcomes through 31 December 2019. Cox proportional hazards models were used to estimate the risk of all-cause, cardiovascular, and cancer mortality. We conducted a mediation analysis using the counterfactual framework method to estimate how ACAG may be indirectly associated with increased mortality risk through mediators.

**Results:**

A total of 2,309 deaths were documented over 8,161 person-years of follow up, including 659 cardiovascular and 399 cancer deaths. In multivariate analyses, higher ACAG levels had a significant correlation with an increase in all-cause (HR, 1.58; 95% CI, 1.38-1.81; P=0.001), cardiovascular (HR, 1.34; 95% CI, 1.05-1.72; P=0.019), and cancer (HR, 1.41; 95% CI, 1.02-1.96; P=0.018) mortality rates than the controls. Results of the mediation analysis showed that altered levels of C-reactive protein and estimated glomerular filtration rate (eGFR) explained 7.867% and 7.669% of the relation between serum ACAG and all-cause mortality, respectively (all P<0.05). Total cholesterol and HbA1c mediated 15.402% and 14.303% of the associations with cardiovascular mortality, respectively (all P<0.05).

**Conclusions:**

Higher ACAG levels were significantly associated with increased all-cause, cardiovascular, and cancer mortality. Researchers suggest that patients with T2D who control ACAG in a normal state may be at a lower risk of mortality.

## 1 Introduction

The global health problem of type 2 diabetes (T2D) has grown significantly in recent years, with more than 463 million diabetics worldwide and an expected rise to 578 million by 2030 (1). According to the World Health Organization’s prediction, diabetes will become the seventh leading cause of death worldwide by 2030. Therefore, fully identifying the modifiable risk factors for premature mortality in a high-risk T2D population is crucial (2).

Anion gap (AG) is one of the important biochemical indicators for evaluating electrolyte and acid-base balance (3), and high AG has been proven to be associated with mortality and disease progression (4–11). However, research on the relationship between AG and T2D risk has produced mixed results. A 3-year follow-up study of 2723 Chinese participants with normal blood glucose levels found that participants with high serum AGs were more likely to experience abnormal glucose metabolism (12). In addition, research using NHANES survey data from the United States found that high AGs are independently associated with higher insulin resistance (13). AG and acid-base balance are critical for maintaining normal physiological states and are related to the occurrence and prognosis of various diseases.

One limitation of using the AG as a diagnostic and prognostic tool is its sensitivity to albumin levels. Albumin, a major plasma protein, significantly influences AG calculation, as hypoalbuminemia can lead to underestimations of true AG levels. This underestimation can mask the presence of metabolic acidosis, leading to misdiagnosis and inappropriate treatment strategies. The albumin-corrected anion gap (ACAG) addresses this issue by adjusting for the albumin concentration, providing a more accurate reflection of the patient’s acid-base status. Studies have shown that ACAG is a more reliable indicator in clinical settings, especially for patients with conditions like diabetes, where albumin levels can frequently be abnormal. By accounting for albumin levels, ACAG reduces the risk of diagnostic errors and provides better insights into the patient’s metabolic state, thereby improving the prediction of adverse outcomes such as mortality. Therefore, incorporating ACAG in clinical practice enhances the accuracy of metabolic assessments and supports more effective management of T2D patients.

Therefore, we aim to study the effect of serum ACAG on all-cause mortality and specific causes of mortality in a nationally representative sample of the United States patients with diabetes. To provide more effective prevention and treatment strategies for clinical practice.

## 2 Methods

### 2.1 Study population

**Figure 1:**
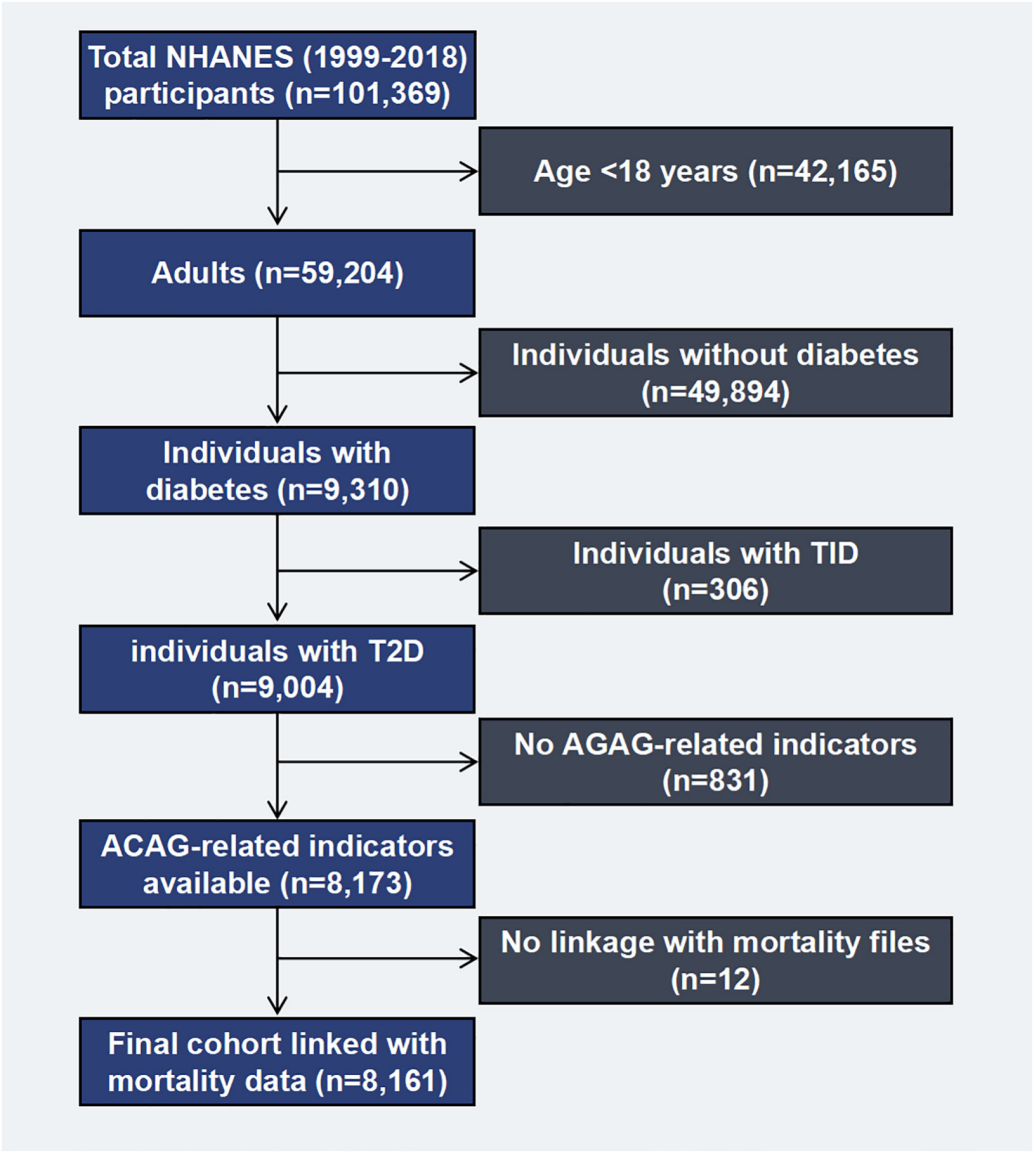
Flow chart of the study population. NHANES, National Health and Nutrition Examination Survey. T1D, type 1 diabetes; T2D, type 2 diabetes; ACAG, Albumin-corrected serum anion gap.

This study was conducted based on NHANES data established by the National Center for Health Statistics (NCHS). By depending on a design of multi-stage probability, non-institutionalized civilian representative samples were obtained through an examination of cross-sectional data. As part of the NHANES exam, the non-institutionalized civilian will be asked questions about their health and nutrition, as well as undergo a physical examination and lab tests. There are other publications that provide details about the study (14–16). The research ethics review committee for NCHS gave its approval to the NHANES study. From 1999 to 2018, survey interviews and physical examination data from the continuous NHANES (n=101,369) were analyzed. A total of 59,204 adults aged 18 years and older were included, while 42,165 adolescent subjects were excluded. Herein, diabetes was defined by one of the following: medication or insulin use, self-reported diagnosis by a doctor, a 7.0 mmol/L fasting glucose level, 6.5% glucose level, or 11.1 mmol/L glucose level after oral glucose tolerance testing, where individuals with T1D (n=306), defined as insulin use and diabetes-onset age at 30 years, were excluded (97% of cases were verified as accurate). T2D was considered in the remaining 9,004 individuals (17). A total of 8,161 diabetic patients with mortality information were measured for serum AG (**Figure 1**).

### 2.2 Measurement of ACAG

For measuring serum sodium ion, chloride ion, bicarbonate, and albumin concentrations, we used Hitachi Mode 1917 multichannel analyzer (Roche Diagnostics, Indianapolis, IN) in the NHANES 1999-2001; Beckman Synchron LX20 (Beckman Coulter Inc., Brea, CA) in 2002-2006; both Beckman Synchron LX 20 and UniCel DxC800 Synchron (Beckman Coulter Inc.) in 2007-2008; Beckman UniCel DxC800 Synchrons (Beckman Coulter Inc.) in 2009-2014; both Beckman UniCel DxC 800 Synchron and UniCel DxC660i Synchron Access chemistry analyzer (Beckman Coulter Inc.) in 2015-2016; the Roche Cobas 6000 Chemistry Analyzer (Roche Diagnostics Corporation, Indianapolis, IN) in 2017-2018. While measuring serum sodium, chloride, bicarbonate, and albumin concentrations, these instruments were used in various ways. The AG was corrected as follows: ACAG (mmol/L) = AG (mmol/L) + (40- [albumin]) ×0.25, with albuminemia in g/L. AG = Na^+^−(Cl^-^ + HCO_3_^-^) (18).

### 2.3 Outcome ascertainment

The critical outcomes in this study are all-cause, cardiovascular, and cancer mortality.

Mortality data up to December 31, 2019 was calculated based on the National Death Index (NDI), in which the death certificates contain the following information. Participants were asked for the name, social security number, race/nationality, birth date and status, gender, and the place where you live. Death causes were classified according to the tenth revision of the International Classification of Diseases. **Supplementary Table 1** illustrates the specific classification of causes of death and their ICD codes.

### 2.4 Covariant evaluation

Individuals self-report their race, age, income, education, and physical activity levels, as well as drug use, diabetes, and cardiovascular disease (CVD) status (utilizing the Rose angina questionnaire). The participant measurements taken during the medical examinations were weight, height at standing, in addition to waist circumference. Calculating the body mass index (BMI) of every participant was performed by division of their body weight (Kg) by height (m^2^). Regarding race, participants were non-Hispanic white people; non-Hispanic black people; Mexican Americans, as well as other Hispanics, Asians, and multiracials. The term “alcohol consumption” refers to drinking alcohol on a regular basis over the preceding year (yes or no). Education can be divided into three levels: below high school, after high school, and college level. According to self-reported income, poverty line, family size, and calendar year, each family’s poverty-income ratio (PIR) is calculated. Those with incomes below the official poverty threshold will have PIR values < 1, while those with incomes above will have PIR values > 1 (range 0-5). Determining physical activity deficiency during a week as never working out, moderate, or high-intensity, whereas physical activity during a week was considered to be moderate or high-intensity where these activities included (walking, jogging, running, swimming, cycling, dancing, or yardwork). The interviewer verified the use of prescription medications by checking the drug container in addition to self-reporting. Patients with hypertension must have an established medical history of measured systolic or diastolic blood pressures above 140 or 90 mmHg, respectively, or self-report being on hypertensive medication. CVD is diagnosed when doctors or other health professionals diagnose participants with congestive heart failure, coronary heart disease, angina pectoris, heart attack, or stroke. Based on the Chronic Kidney Disease Epidemiology Collaboration Study equation, glomerular filtration rate (eGFR) was calculated, and chronic kidney disease (CKD) was determined by eGFR < 60 mL/min/1.73m^2^ (19). Based on the formula, Homeostasis Model Assessment for Insulin Resistance (HOMA-IR) is equal to fasting plasma insulin (μU/L) × fasting blood glucose (mmol/L)/22.5.

### 2.5 Statistical analysis

Because of NHANES complex sampling design, all analyses take into account the weight of research visits, the main sampling units, and the hierarchical design (15, 16). Data analysis was performed using R packages (Version 4.4.1), Stata (Version 17) and IBM SPSS (Version 24) statistical software. Means and standard deviations (SDs) were utilized for describing continuous variables, using the one-way ANOVA and Kruskal-Wallis tests for normal and skewed continuous variables, respectively, while proportions were for categorical variables, and the Chi-square test was applied. A generalized linear model was utilized for examining the correlation between ACAG levels and cardiometabolic biomarkers (insulin, plasma glucose, HOMA-IR, HbA1c, total cholesterol: high-density lipoprotein [HDL], low-density lipoprotein [LDL], triglycerides, urine albumin/creatinine, C-reactive protein [CRP] and eGFR) at baseline. BMI, education, physical activity, PIR, diabetes duration, HbA1c, drug use, CKD, and self-reported disorders were adjusted in order to adjust for the ACAG-mortality correlation potential confounders. Mortality rates were estimated for each ACAG layer/1000 person-years using a Poisson distribution. The ACAG level and all-cause, cardiovascular, and cancer mortality association was calculated using the Cox proportional hazard regression model. Through step-by-step adjustment of risk factors, we tested our models from 1 to 3. In the first model, age, gender, race, and ethnicity were the variables adjusted (Mexican Americans, non-Hispanic black people, non-Hispanic white people, or others). A further adjustment is made for certain factors in Model 2, such as the BMI of individuals, their drinking status, the level of education of the individual, their smoking status, the situation of sports activities, and the relative poverty of family. Additionally, model 3 was adjusted for diabetes drug use, diabetes duration, HbA1c, self-reported disorders (hypertension, congestive heart failure, coronary heart disease, angina, heart attack, stroke), and CKD. With 5.5 mEq/L as a reference value, the association between levels of ACAG and all endpoints was assessed using restricted cubic spline (RCS) curves (four knots). In the mediation analysis, 1000 times permutations and bootstrapping were used. Version 4.4.1 of the R package ‘mediation’ was utilized for estimating the CI. The outcome and mediating variables were characterized based on the generalized and calculated linear model coefficients, respectively.

## 3 Results

### 3.1 Characteristics at baseline

**Table 1** presents the interquartile ranges of ACAG as well as demographic characteristics and confounding factors. In NHANES (1999-2018), data related to ACAG were measured in 8161 T2D participants aged 18 years or older. The participants had an average age of 63 (interquartile range [IQR]: 53-72), and 52.0% of them were men. The majority of participants with higher ACAG levels were women, non-Hispanic white people, alcohol consumers, obese, diabetic for more than ten years, and hypertensive (all P<0.05).

**Table 1:** Baseline characteristics of NHANES participants with diabetes according to serum ACAG.

Characteristics		Serum ACAG (mEq/L)*				*p* value
Number of patients	Total (N=8161)	≤9.50 (n=2191)	9.51-11.00 (n=1988)	11.01-12.75 (n=1953)	≥12.76 (n=2029)	
Demographics and Clinical Characteristics						
Age, years	63 [53,72]	63 [53,71]	64 [53,73]	63 [52,72]	64 [54,73]	0.039
Male (%)	4240 (51.954)	1259 (57.462)	1020 (51.308)	982 (50.282)	979 (48.250)	<0.001
Race/ethnicity						0.001
Non-Hispanic white	2958 (36.246)	734 (33.501)	715 (35.966)	730 (37.378)	779 (38.393)	
Non-Hispanic black	1975 (24.200)	537 (24.509)	480 (24.145)	511 (26.165)	447 (22.031)	
Mexican American	1692 (20.733)	468 (21.360)	434 (21.831)	361 (18.484)	429 (21.143)	
Other	1536 (18.821)	452 (20.630)	359 (18.058)	351 (17.972)	374 (18.433)	
BMI, kg/m^2^	30.83 [26.97,35.89]	30.2 [26.46,34.52]	30.71 [26.93,35.3]	31.29 [27.29,36.72]	31.3 [27.5,37.28]	<0.001
Drinking status						0.17
< 12 drinks/yr	2587 (35.580)	743 (36.764)	662 (36.514)	616 (35.180)	566 (33.571)	
≥ 12 drinks/yr	4684 (64.420)	1278 (63.236)	1151 (63.486)	1135 (64.820)	1120 (66.429)	
Cotinine, ng/mL	0.038 [0.011,0.704]	0.036 [0.011,0.386]	0.036 [0.011,0.661]	0.044 [0.015,1.68]	0.04 [0.011,0.704]	0.003
Education levels						0.126
Less than high school	3078 (37.785)	849 (38.803)	730 (36.757)	767 (39.333)	732 (36.202)	
High school or equivalent	1865 (22.895)	512 (23.400)	457 (23.011)	410 (21.026)	486 (24.036)	
Greater than high school	3203 (39.320)	827 (37.797)	799 (40.232)	773 (39.641)	804 (39.763)	
Family poverty-income ratio	1.8 [1.03,3.4]	1.890 [1.03,3.54]	1.790 [1.07,3.4]	1.810 [1.030,3.41]	1.73 [1.02,3.16]	0.127
Leisure time physical activity						0.494
Never	4481 (54.914)	1190 (54.313)	1114 (56.036)	1041 (53.303)	1136 (56.016)	
Moderate	1758 (21.544)	472 (21.543)	425 (21.378)	427 (21.864)	434 (21.400)	
Vigorous	1921 (23.542)	529 (24.144)	449 (22.586)	485 (24.834)	458 (22.584)	
Duration of diabetes						<0.001
< 3 years	3624 (44.647)	1049 (48.009)	908 (46.021)	862 (44.342)	805 (39.950)	
3-10 years	2203 (27.141)	590 (27.002)	557 (28.231)	525 (27.006)	531 (26.352)	
> 10 years	2290 (28.212)	546 (24.989)	508 (25.748)	557 (28.652)	679 (33.697)	
Medication use						<0.001
No insulin or pills	1352 (16.567)	424 (19.352)	327 (16.449)	305 (15.617)	296 (14.588)	
Only diabetes pills	3668 (44.945)	963 (43.953)	900 (45.272)	880 (45.059)	925 (45.589)	
Any insulin use	1372 (16.812)	292 (13.327)	313 (15.744)	340 (17.409)	427 (21.045)	
Other	1769 (21.676)	512 (23.368)	448 (22.535)	428 (21.915)	381 (18.778)	
HbA1c	6.7 [6.1,7.8]	6.6 [6.0,7.4]	6.7 [6.1,7.8]	6.8 [6.2,8.0]	6.9 [6.3,8.2]	<0.001
eGFR	84.433 [64.409,99.554]	86.799 [69.721,99.714]	84.586 [64.863,99.237]	83.339 [62.635,100.295]	82.301 [59.548,98.945]	<0.001
Self-reported diseases						
Hypertension	5844 (71.626)	1466 (66.910)	1402 (70.523)	1461 (74.846)	1515 (74.704)	<0.001
Congestive heart failure	732 (9.064)	133 (6.126)	163 (8.291)	204 (10.543)	232 (11.577)	<0.001
Coronary heart disease	820 (10.186)	202 (9.304)	203 (10.357)	209 (10.868)	206 (10.321)	0.403
Angina	583 (7.234)	136 (6.250)	131 (6.677)	151 (7.828)	165 (8.283)	0.04
Heart attack	853 (10.519)	195 (8.941)	214 (10.803)	222 (11.485)	222 (11.023)	0.038
Stroke	704 (8.674)	161 (7.379)	165 (8.342)	175 (9.021)	203 (10.069)	0.017

Data were reported as number (percentage), mean (SD), or median (interquartile range [IQR]) when not normally distributed. All estimates accounted for complex survey designs. BMI, body mass index.

### 3.2 Cardiometabolic markers: least squares mean

Using ACAG as a basis, **Table 2** shows the least cardiometabolic biomarker square means. It was found that higher levels of ACAG had a significant correlation to hyperglycemia, insulin, HOMA-IR, HbA1c, total cholesterol, triglycerides, CRP, urine albumin/creatinine and eGFR at baseline (all P<0.05).

**Table 2:** Least squares means of cardiometabolic markers according to serum ACAG concentrations among patients with diabetes in NHANES 1999–2018.

Characteristics	Serum ACAG (mEq/L)				*p* value
	≤9.50 (n=2191)	9.51 – 11.00 (n=1988)	11.01 – 12.75 (n=1953)	≥12.76 (n=2029)	
Glucose (n=4438), mmol/L	7.77 ± 0.10	8.41 ± 0.11	8.61 ± 0.11	9.31 ± 0.12	<0.001
Insulin (n=4413), pmol/L	17.72 ± 0.93	21.62 ± 0.99	21.55 ± 1.01	25.39 ± 1.11	<0.001
HOMA-IR (n=4403)	6.31 ± 0.40	8.15 ± 0.43	8.52 ± 0.44	10.93 ± 0.48	<0.001
HbA1c (n=8147), %	6.88 ± 0.04	7.20 ± 0.04	7.40 ± 0.04	7.50 ± 0.05	<0.001
Total cholesterol (n=8159), mmol/L	4.79 ± 0.03	4.90 ± 0.03	5.00 ± 0.03	5.15 ± 0.03	<0.001
HDL (n=8158), mmol/L	1.24 ± 0.01	1.26 ± 0.01	1.25 ± 0.01	1.25 ± 0.01	0.761
LDL (n=4097), mmol/L	2.79 ± 0.03	2.81 ± 0.03	2.85 ± 0.04	2.82 ± 0.04	0.685
Triglyceride (n=4405), mmol/L	1.67 ± 0.06	1.85 ± 0.06	1.98 ± 0.07	2.32 ± 0.07	<0.001
CRP (n=6355), mg/L	4.21 ± 0.31	5.36 ± 0.32	6.78 ± 0.31	8.61 ± 0.30	<0.001
Urine albumin/creatinine (n=7924), mg/g	65.80 ± 16.38	104.13 ± 17.42	165.43 ± 17.78	257.27 ± 18.61	<0.001
eGFR (n=8159), mL/min per 1.73 m^2^	84.09 ± 0.58	82.32 ± 0.62	80.95 ± 0.63	78.39 ± 0.65	<0.001

### 3.3 Mortality causes and rates

A total of 2309 patients died in the median follow-up period of 7.0 (IQR, 3.1-11.3) years. The cause of 659 deaths was CVD, while the cause of 399 deaths was malignancy. Death rates per 1000 person-years were highest when ACAG levels were in the highest quartile. Among all causes of mortality, 48.54 persons per 1,000 person-years died (95% CI, 45.03-52.31), 14.05 individuals per 1,000 person-years died from CVD causes (95% CI, 12.22-16.15), and 7.02 individuals died from cancer (95% CI, 5.77-8.55). **Table 3** shows the rates of overall and cause-specific mortality/1000 person-years in layer ACAG.

**Table 3:** Causes of death and mortality rates per 1000 person-years across serum ACAG strata.

	Serum ACAG (mEq/L)			
	≤9.50 (n=2191)	9.51 - 11.00 (n=1988)	11.01 - 12.75 (n=1953)	≥12.76 (n=2029)
Cause of death	Mortality rate per 1000 person-years (95% CI)			
All cause deaths	26.23 (23.99-28.68)	32.65 (30.01-35.52)	40.34 (37.24-43.69)	48.54 (45.03-52.31)
Cardiovascular diseases	7.89 (6.71-9.28)	9.35 (7.99-10.95)	10.79 (9.25-12.59)	14.05 (12.22-16.15)
Malignancy	5.39 (4.43-6.56)	5.97 (4.91-7.27)	6.83 (5.63-8.30)	7.02 (5.77-8.55)
Chronic lower respiratory diseases	1.05 (0.66-1.66)	1.50 (1.00-2.24)	2.47 (1.76-3.47)	2.32 (1.60-3.35)
Accidents (unintentional injuries)	0.64 (0.36-1.15)	0.72 (0.40-1.28)	0.67 (0.35-1.28)	0.66 (0.34-1.31)
Cerebrovascular disease	1.92 (1.37-2.70)	1.95 (1.37-2.78)	2.77 (2.01-3.82)	3.31 (2.43-4.50)
Alzheimer disease	0.82 (0.49-1.37)	1.11 (0.69-1.77)	1.20 (0.74-1.95)	1.24 (0.75-2.05)
Diabetes mellitus	2.45 (1.81-3.31)	3.12 (2.35-4.14)	4.72 (3.69-6.04)	5.46 (4.29-6.94)
Influenza and pneumonia	0.47 (0.24-0.92)	0.78 (0.45-1.36)	1.12 (0.68-1.85)	1.24 (0.75-2.05)
Kidney disease	0.64 (0.36-1.15)	1.24 (0.79-1.93)	1.27 (0.79-2.04)	2.98 (2.15-4.12)

CI, 95% confidence interval.

### 3.4 Relationship between ACAG levels and all-cause mortality

**Figure 2:**
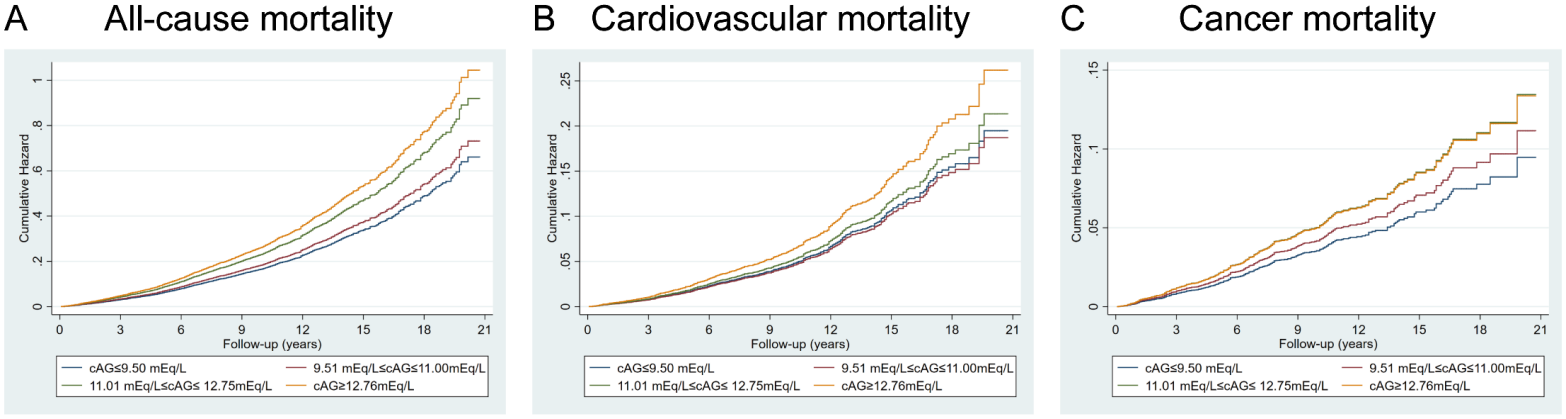
Full multivariable model adjusted survival curve for the target results by baseline albumin-corrected serum anion gap (ACAG). **(A)** All-cause mortality. **(B)** Cardiovascular mortality. **(C)** Cancer mortality. Cox regression model with complex investigation design was used for analysis. HRs were adjusted according to age (continuous), sex (male, or female) and race/ethnicity (non-Hispanic white people, Non-Hispanic black people, Mexican American or other), BMI (continuous), drinking status (< 12 drinks/yr, ≥ 12 drinks/yr), cotinine (continuous), education level (less than high school, high school or equivalent, or greater than high school), family poverty-income ratio (continuous), physical activity (never, moderate or vigorous), duration of diabetes (< 3, 3–10, or > 10 years), and diabetes medication use (none, oral medication and/or insulin), HbA1c (continuous), self-reported diseases (hypertension, congestive heart failure, coronary heart disease, angina, heart attack, stroke), eGFR.

**Figure 3:**
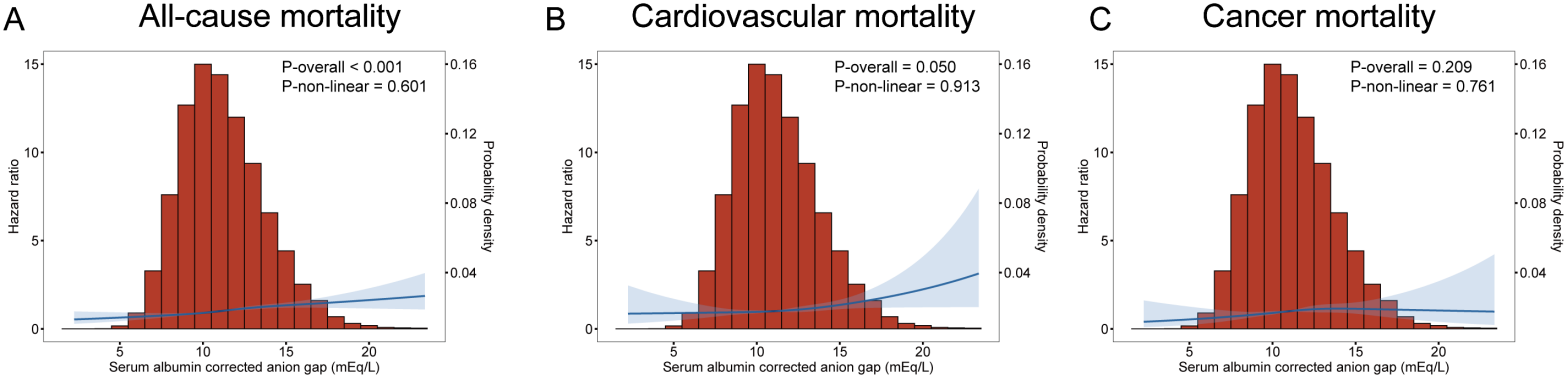
Multivariate adjusted smoothing spline plots of ACAG levels with all-cause **(A)**, cardiovascular **(B)** and cancer **(C)** mortality among T2D patients in NHANES 1999-2018. HRs were adjusted according to age (continuous), sex (male, or female) and race/ethnicity (non-Hispanic white people, Non-Hispanic black people, Mexican American or other), BMI (continuous), drinking status (< 12 drinks/yr, ≥ 12 drinks/yr), cotinine (continuous), education level (less than high school, high school or equivalent, or greater than high school), family poverty-income ratio (continuous), physical activity (never, moderate or vigorous), duration of diabetes (< 3, 3–10, or >10 years), and diabetes medication use (none, oral medication and/or insulin), HbA1c (continuous), self-reported diseases (hypertension, congestive heart failure, coronary heart disease, angina, heart attack, stroke), eGFR. Both p linearity<0.001. The blue curve represents the best fit line, and the light blue curve represents 95% confidence interval.

Multivariate analysis shows ACAG levels to be associated with mortality in **Figure 2**. Cox proportional hazard models revealed an increased risk for all-cause mortality associated with ACAG levels (HR, 1.58; 95% CI, 1.38-1.81; P=0.001) relative to Q1, in the multivariable-adjusted model (**Table 4**). The RCS plot demonstrates a significant linear association between ACAG and all-cause mortality, without an apparent cut point (**Figure 3A**).

**Table 4:** Cox regression models for the association between serum ACAG groups and clinical outcomes accounting for complex survey design.

	Serum ACAG (mEq/L)						
	≤9.50 (n=2191)	9.51 - 11.00 (n=1988)	11.01 - 12.75 (n=1953)	≥12.76 (n=2029)	P-trend	1-SD increase in ACAG	P value
All-cause mortality							
No. deaths	482	541	602	684			
Model 1	Ref	1.20 (1.06-1.36); 0.003	1.53 (1.36-1.73); <0.001	1.80 (1.60-2.03); <0.001	<0.001	1.05 (1.03-1.07)	<0.001
Model 2	Ref	1.16 (1.01-1.33); 0.039	1.52 (1.33-1.74); <0.001	1.76 (1.54-2.02); <0.001	<0.001	1.06 (1.04-1.08)	<0.001
Model 3	Ref	1.11 (0.96-1.27); 0.156	1.39 (1.21-1.60); <0.001	1.58 (1.38-1.81); <0.001	<0.001	1.04 (1.02-1.07)	<0.001
Cardiovascular mortality							
No. deaths	145	155	161	198			
Model 1	Ref	1.14 (0.91-1.44); 0.244	1.36 (1.09-1.71); 0.007	1.72 (1.38-2.14); <0.001	<0.001	1.05 (1.02-1.08)	0.002
Model 2	Ref	1.01 (0.78-1.29); 0.956	1.23 (0.96-1.57); 0.101	1.52 (1.19-1.93); 0.001	<0.001	1.06 (1.02-1.10)	0.007
Model 3	Ref	0.96 (0.75-1.24); 0.754	1.10 (0.85-1.41); 0.477	1.34 (1.05-1.72); 0.019	0.011	1.03 (0.99-1.08)	0.148
Cancer mortality							
No. deaths	99	99	102	99			
Model 1	Ref	1.10 (0.83-1.46); 0.498	1.32 (1.00-1.74); 0.053	1.35 (1.01-1.79); 0.039	0.018	1.03 (0.98-1.09)	0.279
Model 2	Ref	1.16 (0.85-1.58); 0.344	1.39 (1.02-1.89); 0.035	1.37 (1.00-1.89); 0.053	0.025	1.04 (0.98-1.11)	0.187
Model 3	Ref	1.18 (0.86-1.61); 0.298	1.42 (1.04-1.94); 0.027	1.41 (1.02-1.96); 0.038	0.018	1.05 (0.98-1.11)	0.147

Model 1 is adjusted for age (continuous), sex (male, or female) and race/ethnicity (non-Hispanic white, non-Hispanic black, Mexican American or other).

Model 2 is adjusted for variables in Model 1+BMI (continuous), drinking status (<12 drinks/yr; ≥12 drinks/yr), Cotinine (continuous), education level (less than high school, high school or equivalent, or greater than high school), family poverty-income ratio (continuous), physical activity (never, moderate or vigorous).

Model 3 is adjusted for variables in Model 2 + duration of diabetes (<3, 3–10, or.>10 years), and diabetes medication use (none, oral medication and/or insulin). HbA1c (continuous), self-reported diseases (Hypertension, congestive heart failure, coronary heart disease, angina, heart attack, stroke), eGFR.

**Figure 4:**
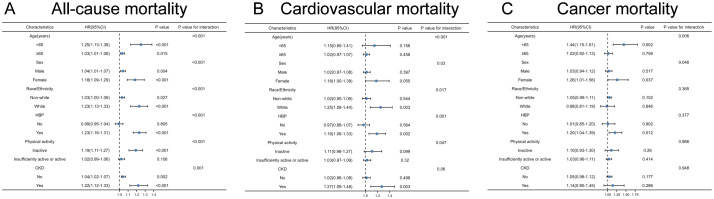
Albumin-corrected serum anion gap (ACAG) levels with all-cause **(A)**, cardiovascular **(B)** and cancer **(C)** mortality in subgroups. HRs were adjusted according to age (continuous), sex (male, or female) and race/ethnicity (non-Hispanic white people, Non-Hispanic black people, Mexican American or other), BMI (continuous), drinking status (< 12 drinks/yr, ≥ 12 drinks/yr), cotinine (continuous), education level (less than high school, high school or equivalent, or greater than high school), family poverty-income ratio (continuous), physical activity (never, moderate or vigorous), duration of diabetes (< 3, 3-10, or >10 years), and diabetes medication use (none, oral medication and/or insulin), HbA1c (continuous), self-reported diseases (hypertension, congestive heart failure, coronary heart disease, angina, heart attack, stroke), eGFR. P values are for interaction between subgroup and ACAG levels. CKD, chronic kidney disease.

As a result of the subgroup analysis, **Figure 4** presents the forest plots. All-cause mortality was higher in younger adults, women, and white people compared to the normal group in subgroup analyses (all P<0.05) (**Figure 4A**). A higher ACAG at baseline was correlated to a 22% and 23% higher all-cause mortality risk, respectively, among CKD participants (HR, 1.22; 95% CI, 1.12-1.33; P<0.001) and hypertension (HR, 1.23; 95% CI, 1.16-1.31; P<0.001).

### 3.5 Relationship between ACAG level and cardiovascular mortality

Cardiovascular death rates were significantly higher in participants with serum ACAG above 12.76 mEq/L (**Figure 2B**). A Cox regression analysis was used for determining classification predictors for ACAG. Following the variable adjustment in model 3, patients with the highest quartile ACAG had a significantly increased cardiovascular mortality risk than in the reference category (HR, 1.34; 95% CI, 1.05–1.72; P=0.019) (**Table 4**). **Figure 3B** illustrates that ACAG has a linear relationship to cardiovascular mortality after multivariable adjustment.

Compared to the other group, people who were White had a higher chance of increasing cardiovascular mortality (**Figure 4B**). A high ACAG level at baseline, more than 12.76 mEq/L, was correlated to high cardiovascular mortality in the CKD subgroup (HR, 1.27; 95% CI, 1.09-1.48; P=0.003).

### 3.6 Relationship between ACAG level and cancer mortality

Participants with a ACAG above 12.76 mEq/L had a 41% elevated cancer mortality rate than the Q1 group after multivariate (lifestyle factors, BMI, diabetes duration, diabetes drug use, and chronic disorders) analysis and adjustment (HR, 1.41; 95% CI, 1.02-1.96; P=0.018) (**Figure 2C**, **Table 4**). The spline of ACAG suggested neither a linear nor a non-linear relationship between ACAG and cancer mortality (**Figure 3C**).

The ACAG was higher among younger people, which increased cancer mortality (HR, 1.44; 95% CI, 1.15-1.81; P=0.002) (**Figure 4C**). ACAG levels above 12.76 mEq/L had a correlation to a high cancer mortality risk than the other group (HR, 1.20; 95% CI, 1.04-1.39; P=0.012).

### 3.7 Mediation analysis

**Table 5** summarizes the results of our mediation analysis, which found that CRP and eGFR were significant mediators in the association between serum ACAG and all-cause mortality, explaining 7.867% and 7.669% of the relation, respectively. Additionally, altered levels of total cholesterol and HbA1c significantly mediated the relationship between serum ACAG and CVD mortality, explaining 15.402% and 14.303% of the associations, respectively. All of these results were statistically significant (P <0.05).

**Table 5:** Mediation analysis for metabolic, cardiovascular, and renal markers as possible mediators of the association of serum ACAG with clinical outcomes among individuals ^a^.

	Total effect				Direct effect				Indirect effect				% mediated b
	OR	Lower 95% CI	Upper 95% CI	P-value	OR	Lower 95% CI	Upper 95% CI	P-value	OR	Lower 95% CI	Upper 95% CI	P-value	
All-cause mortality													
Glucose, mmol/L	1.0117	1.0108	1.0124	<0.001	1.0112	1.0103	1.0120	<0.001	1.0005	1.0002	1.0009	<0.001	4.297%
Insulin, pmol/L	1.0117	1.0109	1.0123	<0.001	1.0117	1.0108	1.0124	<0.001	1.0000	0.9998	1.0002	0.9640	0.000%
HOMA-IR	1.0116	1.0107	1.0124	<0.001	1.0116	1.0106	1.0124	<0.001	1.0000	0.9998	1.0003	0.7160	0.000%
HbA1c, %	1.0117	1.0111	1.0123	<0.001	1.0111	1.0102	1.0117	<0.001	1.0006	1.0003	1.0011	<0.001	5.157%
Total cholesterol, mmol/L	1.0116	1.0107	1.0122	<0.001	1.0108	1.0098	1.0115	<0.001	1.0008	1.0005	1.0011	<0.001	6.934%
HDL, mmol/L	1.0116	1.0107	1.0122	<0.001	1.0116	1.0107	1.0122	<0.001	1.0000	0.9999	1.0000	0.6940	0.000%
LDL, mmol/L	1.0110	1.0095	1.0119	<0.001	1.0110	1.0095	1.0119	<0.001	1.0000	0.9999	1.0002	0.5700	0.000%
Triglyceride, mmol/L	1.0118	1.0110	1.0125	<0.001	1.0113	1.0103	1.0121	<0.001	1.0005	1.0001	1.0011	0.0220	4.261%
CRP, mg/L	1.0115	1.0095	1.0129	<0.001	1.0106	1.0085	1.0121	<0.001	1.0009	1.0004	1.0014	<0.001	7.867%
Urine albumin/creatinine, mg/g	1.0113	1.0104	1.0120	<0.001	1.0110	1.0101	1.0117	<0.001	1.0003	1.0001	1.0005	0.0060	2.669%
eGFR, mL/min per 1.73 m^2^	1.0118	1.0111	1.0123	<0.001	1.0109	1.0101	1.0115	<0.001	1.0009	1.0006	1.0012	<0.001	7.669%
Cardiovascular mortality													
Glucose, mmol/L	1.0029	1.0014	1.0036	0.0060	1.0027	1.0011	1.0034	0.0060	1.0002	1.0000	1.0005	0.1100	6.906%
Insulin, pmol/L	1.0029	1.0016	1.0035	0.0020	1.0030	1.0016	1.0036	0.0020	0.9999	0.9998	1.0000	0.2160	-3.453%
HOMA-IR	1.0029	1.0014	1.0036	0.0060	1.0030	1.0014	1.0036	0.0060	0.9999	0.9998	1.0001	0.4220	-3.453%
HbA1c, %	1.0028	1.0019	1.0033	<0.001	1.0024	1.0013	1.0031	<0.001	1.0004	1.0001	1.0007	0.0020	14.303%
Total cholesterol, mmol/L	1.0026	1.0013	1.0033	<0.001	1.0023	1.0009	1.0030	0.0100	1.0004	1.0002	1.0006	<0.001	15.402%
HDL, mmol/L	1.0026	1.0014	1.0032	<0.001	1.0026	1.0014	1.0032	<0.001	1.0000	0.9999	1.0000	0.6400	0.000%
LDL, mmol/L	1.0026	1.0011	1.0034	0.0100	1.0026	1.0010	1.0034	0.0100	1.0000	0.9999	1.0002	0.5620	0.000%
Triglyceride, mmol/L	1.0030	1.0017	1.0036	<0.001	1.0028	1.0014	1.0034	<0.001	1.0002	1.0000	1.0004	0.0520	6.676%
CRP, mg/L	1.0025	1.0007	1.0034	0.0160	1.0024	1.0006	1.0033	0.0200	1.0001	0.9999	1.0004	0.2820	4.005%
Urine albumin/creatinine, mg/g	1.0027	1.0017	1.0032	<0.001	1.0026	1.0016	1.0032	<0.001	1.0001	1.0000	1.0002	0.1960	3.709%
eGFR, mL/min per 1.73 m^2^	1.0028	1.0018	1.0033	0.0020	1.0025	1.0014	1.0031	0.0020	1.0003	1.0001	1.0005	<0.001	10.728%
Cancer mortality													
Glucose, mmol/L	1.0010	0.9982	1.0023	0.2820	1.0010	0.9981	1.0023	0.2820	1.0000	0.9997	1.0003	0.9800	0.000%
Insulin, pmol/L	1.0012	0.9986	1.0022	0.2080	1.0012	0.9986	1.0023	0.2180	1.0000	0.9999	1.0001	0.9700	0.000%
HOMA-IR	1.0011	0.9981	1.0023	0.2680	1.0011	0.9981	1.0023	0.2820	1.0000	0.9998	1.0003	0.8460	0.000%
HbA1c, %	1.0012	0.9997	1.0020	0.0840	1.0016	1.0002	1.0023	0.0320	0.9997	0.9993	1.0000	0.0540	-25.019%
Total cholesterol, mmol/L	1.0015	1.0002	1.0020	0.0320	1.0013	1.0001	1.0019	0.0460	1.0001	1.0000	1.0003	0.1200	6.671%
HDL, mmol/L	1.0014	1.0001	1.0020	0.0340	1.0014	1.0001	1.0020	0.0360	1.0000	1.0000	1.0001	0.5540	0.000%
LDL, mmol/L	1.0008	0.9969	1.0022	0.4280	1.0007	0.9969	1.0022	0.4360	1.0000	1.0000	1.0001	0.6360	0.000%
Triglyceride, mmol/L	1.0013	0.9988	1.0023	0.1840	1.0012	0.9986	1.0023	0.2060	1.0000	0.9998	1.0003	0.8320	0.000%
CRP, mg/L	1.0007	0.9983	1.0020	0.3600	1.0005	0.9980	1.0018	0.4920	1.0002	1.0000	1.0005	0.0660	28.579%
Urine albumin/creatinine, mg/g	1.0014	1.0001	1.0020	0.0300	1.0015	1.0003	1.0021	0.0260	0.9999	0.9997	1.0001	0.3880	-7.148%
eGFR, mL/min per 1.73 m^2^	1.0014	1.0000	1.0020	0.0520	1.0015	1.0001	1.0021	0.0360	0.9999	0.9997	1.0000	0.1980	-7.148%

^a^Odd ratios were adjusted for age (continuous), sex (male, or female) and race/ethnicity (non-Hispanic white, non-Hispanic black, Mexican American or other), BMI (continuous), drinking status (< 12 drinks/yr, ≥ 12 drinks/yr), cotinine (continuous), education level (less than high school, high school or equivalent, or greater than high school), family poverty-income ratio (continuous), physical activity (never, moderate or vigorous), duration of diabetes (<3, 3–10, or >10 years), and diabetes medication use (none, oral medication and/or insulin). HbA1c (continuous), self-reported diseases (Hypertension, congestive heart failure, coronary heart disease, angina, heart attack, stroke), eGFR. HOMA-IR, Homeostasis Model Assessment for Insulin Resistance; HDL, high density lipoprotein; LDL, low density lipoprotein; CRP, C-reactive protein; eGFR, estimated glomerular filtration rate.

^b^The percentage mediated was calculated by log (indirect effect)/log (total effect).

## 4 Discussion

**Figure 5:**
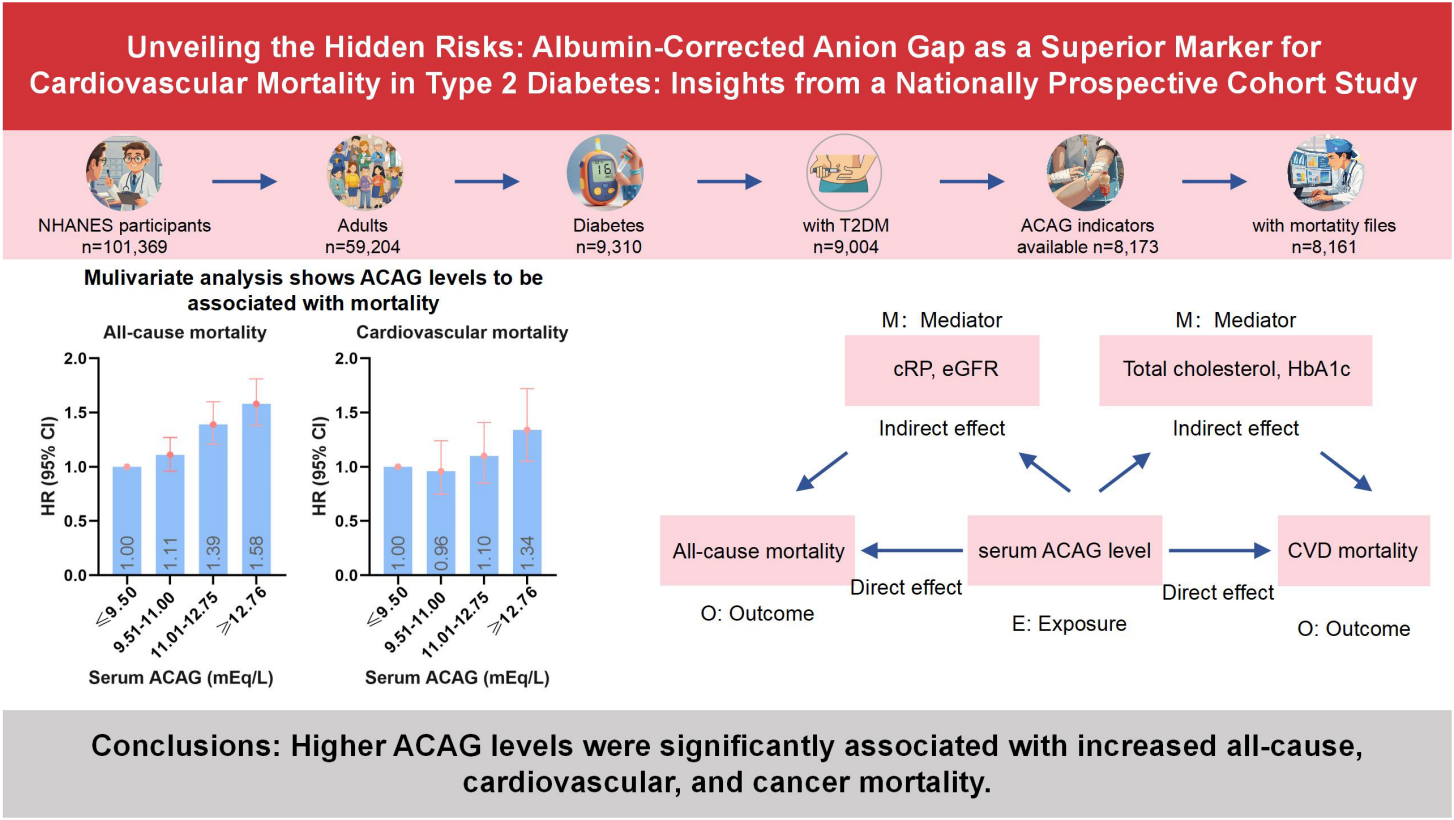
Scheme of the aim of the study and participants selection process.

In a large cohort study of U.S. adults with diabetes, we found that patients with ACAG levels above 12.76 mEq/L had a significantly increased risk of all-cause, cardiovascular, and cancer mortality over an average follow-up of 7 years (**Figure 5**). CRP and eGFR emerged as significant mediators in the relationship between serum ACAG and all-cause mortality, explaining 7.867% and 7.669% of the association, respectively. Additionally, total cholesterol and HbA1c levels significantly mediated the relationship between serum ACAG and CVD mortality, explaining 15.402% and 14.303% of the association, respectively.

The association between AG levels and mortality has been well-documented in multiple studies, though these studies did not specifically target T2D patients. For instance, an analysis of 18,115 patients with coronary heart disease identified increased serum AG as an independent predictor of all-cause mortality (20). Similarly, studies on patients with spontaneous subarachnoid hemorrhage (SAH) and cardiac arrest have shown that higher AG concentrations are associated with increased mortality (10). In addition, a study on AG and mortality in patients with cardiac arrest found that patients with high AG levels are less likely to survive during hospitalization than those with low AG levels (21). It is worth noting that these studies usually do not use albumin to calibrate AG, and the samples are mostly from hospitals and intensive care units. Therefore, when interpreting research results, it is necessary to consider the possible biases caused by these factors. Our study, using a large-scale cohort of T2D patients from the NHANES database, addresses these limitations by including a nationally representative sample covering multiple races, thereby expanding the study’s scope and improving its applicability.

The mechanisms underlying the association between elevated ACAG and mortality are multifaceted. Higher ACAG levels reflect a greater degree of metabolic acidosis, which has been implicated in the development and progression of various complications in T2D. Metabolic acidosis can exacerbate insulin resistance, promote inflammation, and contribute to endothelial dysfunction, thereby increasing the risk of cardiovascular diseases and other complications. Our mediation analysis further supports this by showing that CRP and eGFR significantly mediate the relationship between ACAG and all-cause mortality, while total cholesterol and HbA1c mediate the association with cardiovascular mortality.

Metabolic acidosis mainly manifested by excessive acidic metabolites such as ketones and lactic acid (22). These metabolites can increase the acid load on the kidneys, leading to tubular damage and deterioration of renal function (23–25). At the same time, metabolic acidosis can also lead to electrolyte disorders (26, 27), such as the loss of potassium and calcium ions, which are crucial for maintaining normal kidney function. Therefore, metabolic acidosis will aggravate the renal disease of patients with diabetes, and even cause renal failure.

In addition, patients with diabetes are often accompanied by chronic low-grade inflammation (28, 29). Metabolic acidosis will aggravate this inflammatory reaction, leading to the increase of CRP level in patients (30). Under the condition of high inflammation for a long time, the immune system of diabetes patients is easy to be out of balance, increasing the risk of infection and cardiovascular events. Metabolic acidosis reduces circulating adiponectin level by inhibiting adiponectin gene transcription, and promotes the development of atherosclerosis (31). In addition, metabolic acidosis can also cause oxidative stress and inflammatory reaction in the body, aggravate vascular endothelial cell damage and atherosclerosis and other diseases, lead to an increase in free fatty acids (FFA) level, and increase the risk of cardiovascular disease and death (32).

Glycated hemoglobin is an important indicator of blood sugar control status. Metabolic acidosis can lead to an increase in blood sugar levels (33, 34), accelerate the glycation reaction of hemoglobin, and lead to an increase in glycated hemoglobin levels (35). The elevated level of glycosylated hemoglobin will cause systemic inflammatory reaction and oxidative stress, leading to microvascular and large vascular damage (36–38), aggravating the risk of complications and death of diabetes. In addition, metabolic acidosis will also affect the renal function of patients with diabetes, further aggravating the development of poor blood sugar control and diabetes complications.

Recent studies have shown a certain correlation between metabolic acidosis and an increased risk of cancer death. Metabolic acidosis can cause systemic inflammatory reactions and oxidative stress, thereby affecting immune system function and cellular DNA repair ability, increasing the risk of cancer occurrence and death (39–42). In addition, metabolic acidosis can lead to an increase in the acidic environment in the body, which in turn affects cell growth and metabolism, accelerating the growth and spread of cancer cells (43). Research has shown that metabolic acidosis is associated with an increased risk of occurrence and death from various cancers such as lung cancer (44, 45), colorectal cancer (46, 47), and prostate cancer (48, 49). Diabetes patients themselves have a high incidence of cancer and mortality, and the aggravation of metabolic acidosis may further exacerbate this risk.

A key point raised during the review process is the heterogeneity of the patient population included in this study. The participants had varying comorbid conditions, ranging from cardiovascular disease to malignancy and infections. This variation introduces complexity into the interpretation of our findings. While our primary focus was on the role of ACAG in predicting mortality, it is important to acknowledge that the presence of multiple and diverse pathologies could confound the association between ACAG and mortality outcomes. Different diseases, such as cardiovascular disorders, neoplasms, and infections, have distinct pathophysiological mechanisms, and combining them under a single analysis may obscure condition-specific influences.

The current study utilizes a large samples, and nationally representative samples of American adults with diabetes. In this way, we are able to generalize our findings. Comprehensive NHANES data allowed for adjusting various potential confounding factors, such as race/ethnicity, socioeconomic status, lifestyle variables, diabetes duration, and comorbidities, in addition to diabetes drug use. The study also has multiple constraints. First, the retrospective nature of this study is another limitation that must be acknowledged. Retrospective cohort studies are subject to various biases, including unmeasured confounding and reverse causality, which may limit the ability to draw definitive causal inferences. While we adjusted for numerous confounding factors, residual confounding could still exist. Moreover, the ACAG values were measured at a single time point, and we could not account for longitudinal changes in ACAG or other biochemical markers over time. Third, a detailed description of diabetes severity was not provided in this study. Even after adjusting for diabetes medication use, diabetes duration, eGFR, HbA1c levels, and multiple self-reported comorbidities, the results remain significant. Fourth, mortality outcomes were determined using a probabilistic match, resulting in possible misclassifications. Despite a high degree of accuracy shown in a previous validation study (50). Finally, we cannot rule out confounding resulting from psychological stress, genetic susceptibility, residual confounding, or serendipitous.

## 5 Conclusion

According to a representative nationally-based sample of US adults who were diabetic with higher ACAG were strongly correlated to greater all-cause, cardiovascular, and cancer mortality. Using those results in clinical trials would benefit high-risk patients with high ACAG levels and T2D in the future.

## Data Availability

The original contributions presented in the study are included in the article/**Supplementary Material**. Further inquiries can be directed to the corresponding authors.
