# Supplementary material for: Unveiling the hidden risks: albumin-corrected anion gap as a superior marker for cardiovascular mortality in type 2 diabetes: insights from a nationally prospective cohort study
Source: Front Endocrinol (Lausanne). 2024 Nov 7;15:1461047. doi: 10.3389/fendo.2024.1461047 (PMC11578733; doi:10.3389/fendo.2024.1461047)
Supplement: Supplementary file 1 [file Table1.docx]

Supplemental Table 1. Classification of causes of death and their ICD codes

| Cause of death | ICD codes |
| --- | --- |
| All causes | - |
| Cardiovascular diseases | I00-I09, I11, I13, I20-I51 |
| Chronic lower respiratory diseases | J40-J47 |
| Accidents | V01-X59, Y85-Y86 |
| Cerebrovascular disease | I60-I69 |
| Alzheimer’s disease | G30 |
| Diabetes mellitus | E10-E14 |
| Influenza and pneumonia | J10-J18 |
| Kidney-related diseases | N00-N07, N17-N19, N25-N27 |

This table classifies and numbers the underlying causes of death according to the tenth revision of the International Classification of Diseases.
